# Chemical characterization and antimicrobial activities of *Citrus aurantifolia* peel oils and *Ocimum sanctum* ethanolic extract

**DOI:** 10.1371/journal.pone.0331710

**Published:** 2025-09-05

**Authors:** Pintana Duangsombat, Neti Waranuch, Tasana Pitaksuteepong

**Affiliations:** 1 Department of Pharmaceutical Technology, Faculty of Pharmaceutical Sciences, Naresuan University, Phitsanulok, Thailand; 2 Center of Excellence for Innovation in Chemistry, Naresuan University, Phitsanulok, Thailand; 3 Cosmetics and Natural Products Research Centre (CosNat), Faculty of Pharmaceutical Sciences, Naresuan University, Phitsanulok, Thailand; Universidad Autonoma de Chihuahua, MEXICO

## Abstract

Oral diseases affect more than 3.5 billion people globally, representing a major public health burden, particularly in low- and middle-income countries where access to dental care is often limited. Furthermore, the use of conventional antimicrobial agent may cause side effect. This underscores the need for affordable, plant-based alternatives to conventional antimicrobials. This study investigated the chemical compositions and antimicrobial activities of *Citrus aurantifolia* peel oils from Thailand (Lime TH) and South Africa (Lime SF), along with an ethanolic extract of *Ocimum sanctum* L. (OSE), against five oral pathogens: *Lactobacillus acidophilus*, *Streptococcus mutans*, *Porphyromonas gingivalis*, *Aggregatibacter actinomycetemcomitans*, and *Candida albicans*. Chemical constituents were analyzed using gas chromatography-mass spectrometry (GC-MS) for the peel oils and high-performance liquid chromatography (HPLC) for the extract. GC-MS identified D-limonene as the major constituent in both Lime TH (49.11 ± 0.76% w/w) and Lime SF (42.32 ± 0.60% w/w), while HPLC confirmed the presence of ursolic acid in OSE (2.67 ± 0.07% w/w). Antimicrobial activity was evaluated by broth microdilution to determine minimum inhibitory concentrations (MIC), minimum bactericidal/fungicidal concentrations (MBC/MFC), and time-kill kinetics. Lime TH exhibited the strongest activity (MIC and MBC/MFC values between 0.20 and 25.0 mg/mL), followed by Lime SF (0.39–50.0 mg/mL). OSE inhibited four bacterial strains (excluding *C. albicans*) with MIC and MBC values ranging from 0.05 to 100 mg/mL. These findings highlight the potential of *C. aurantifolia* peel oils and *O. sanctum* extract as natural antimicrobial agents for incorporation into oral care products.

## Introduction

Oral health is a fundamental component of overall well-being and a significant public health concern. Oral diseases affect an estimated 3.5 billion people worldwide, making them the most prevalent health conditions globally—surpassing the combined burden of cardiovascular diseases, cancers, diabetes, chronic respiratory diseases, and mental disorders [1]. The impact is especially severe in low- and middle-income countries, where access to oral healthcare services is often limited. This underscores an urgent need for alternative strategies that are both effective and accessible. Naturally derived compounds have attracted increasing attention due to their antimicrobial activity, natural origin, affordability, and local availability [2].

Dental caries and periodontitis are highly prevalent oral pathologies [3]. Dental caries results from the demineralization of tooth enamel by acids produced through bacterial fermentation of dietary sugars. *Streptococcus mutans* and *Lactobacillus acidophilus* are primary cariogenic organisms [3,4]. *S. mutans*, an anaerobic Gram-positive bacterium, adheres to the dental plaque biofilm and ferments sugars to produce lactic acid, which demineralizes tooth enamel [4]. Key risk factors for dental biofilm formation and caries include high consumption of free sugars, insufficient fluoride exposure, and poor oral hygiene [5]. *Lactobacillus* species, particularly *L. acidophilus*, thrive in acidic, carbohydrate-rich environments, promoting lesion formation and produces biosurfactants that interfere with *S. mutans* biofilm formation by suppressing gtfB and gtfC gene expression [6]. It can also inhibit *Porphyromonas gingivalis* [7] and downregulate virulence factors of *Aggregatibacter actinomycetemcomitans* [8], partly through enzymatic biofilm disruption [9]. Periodontal diseases, which begin as gingivitis and may progression to periodontitis, cause tooth mobility, loss, and impaired mastication [10]. Gingivitis, develops from bacterial biofilm accumulation, leads to gum inflammation and bleeding [11]. Without treatment, plaque can calcify into calculus, creating niches for pathogens such as *P. gingivalis* and *A. actinomycetemcomitans* [12]. These bacteria release virulence factors, including enzymes (collagenases, proteases, peptidyl arginine deiminase, dispersin B), lipopolysaccharides, hydrogen sulfide, and ammonia, that promote connective tissue degradation and alveolar bone resorption [13]. Opportunistic pathogens such as *Candida albicans* may also overgrow, particularly in immunocompromised individuals, causing oral candidiasis [14].

Fluoride and chlorhexidine are common antimicrobial agents in oral care products [15]. While fluoride prevents enamel demineralization, excessive long-term intake may cause dental fluorosis [16,17]. Commercial toothpaste generally contains 1,000–1,500 ppm fluoride [18,19], with each brushing delivering 0.5–1.0 g [20]. Chlorhexidine is highly effective against plaque and gingivitis but can cause tooth staining, altered taste perception, and, with prolonged use, microbial resistance [21]. These limitations drive the search for plant-based antimicrobial agents.

*Citrus aurantifolia* (Lime), belongs to the Rutaceae family. It is widely cultivated in Thailand, particularly in Phetchaburi province. Essential oils extracted from the leaves and fruit peel of *C. aurantifolia* have been reported to contain limonene (77.5%), linalool (20.1%), and citronellal (14.5%), with antimicrobial activity against *S. mutans* (MIC: 20 µg/mL) [22]. The peel oils also showed antifungal activity against *C. albicans* [23]. Ethanolic peel extracts have shown inhibitory effects against *L. acidophilus*, *P. gingivalis*, and *A. actinomycetemcomitans* [24,25], though most studies used crude solvent extracts rather than pure hydrodistilled oils, which differ markedly in chemical composition and potency.

*Ocimum sanctum* (holy basil), another widely cultivated plant in Thailand, has demonstrated antimicrobial effects relevant to oral pathogens. Ethanolic leaf extracts have inhibited *S. mutans* and *L. acidophilus*, with MIC values of 25 mg/mL and 100 mg/mL, respectively [26], and shown activity against *A. actinomycetemcomitans* comparable, though slightly inferior, to 0.2% chlorhexidine [27]. However, most existing studies have used agar diffusion methods without standardized time–kill kinetics or MIC/MBC determination, limiting comparability.

Despite increasing interest in these natural agents, few studies have comprehensively compared the chemical profiles and antimicrobial properties of *C. aurantifolia* peel oil and *O. sanctum* ethanolic extract, particularly against both cariogenic and periodontal pathogens. Moreover, geographical and environmental factors can markedly influence phytochemical composition, necessitating region-specific characterization. This study addresses these gaps by: (i) characterizing the chemical profiles of Thai and South African *C. aurantifolia* peel oils obtained via hydrodistillation; (ii) analyzing *O. sanctum* ethanolic extract; and (iii) evaluating their antimicrobial efficacy, including MIC, MBC/MFC, and time–kill kinetics, against clinically relevant oral pathogens. The findings are expected to offer valuable insights into their potential as alternative agents in oral healthcare products such as mouth rinses, gels, or dentifrices.

## Materials and methods

### Natural agents tested

Two sources of *C. aurantifolia* (lime) peel oils were used: Thai *C. aurantifolia* peel oils (Lime TH) from Siam Worada 59 Co., Ltd. (Phetchaburi, Thailand) and South African *C. aurantifolia* peel oils (Lime SF) from organic farmland in Randburg region (Gauteng, South Africa). Powdered leaves of *O. sanctum* L. (holy basil) were acquired from Vejpong Pharmacy Co., Ltd. (Bangkok, Thailand).

### Culture media, chemicals and reagents

Brain Heart Infusion Broth and Agar, Sabouraud Dextrose Broth and Agar, and Tryptic Soy Broth and Agar were purchased from Difco-Becton Dickinson (Sparks, MD, USA). Lactobacillus MRS Broth and Agar, yeast extract, and L-cysteine hydrochloride were obtained from Hi-media Laboratories (Mumbai, India). Ultrapure water Type I was generated using MilliQ equipment (Merck KGaA, Darmstadt, Germany). Ethanol 95% (USP-grade) was purchased from the Liquor Distillery Organization (Chachoengsao, Thailand). Methanol (HPLC grade) and acetonitrile (HPLC grade) were purchased from RCI Labscan (Bangkok, Thailand). Dimethyl sulfoxide (DMSO), vitamin K1, and hemin were purchased from Sigma-Aldrich (Saint Louis, MO, USA). Tween 80 was purchased from Panreac Applichem (Barcelona, Spain). A 2% w/v chlorhexidine gluconate solution was purchased from Pose Health Care (Bangkok, Thailand). The D-limonene standard solution [(R)-(+)-limonene, 99% purity] and ursolic acid standard solution (97% purity) were purchased from Sigma-Aldrich.

### Determination of chemical compositions of *C. aurantifolia* peel oils and quantitative analysis of D-limonene

The chemical compositions of Lime TH, Lime SF and the quantification of D-limonene were analyzed using gas chromatography–mass spectrometry (GC–MS; Model 8890–5977B GC–MSD, Agilent, Santa Clara, CA, USA) in synchronous Selective Ion Monitoring and Full scan (SIM/SCAN) mode. Full scan mode was used to identify compounds by matching with the NIST Mass Spectral Search program version 2.4 (Gaithersburg, USA) and to calculate the relative peak area of each compound. Then, SIM mode was used to quantitative analysis of target compound (D-limonene) using selected ions. The GC-MS conditions were modified based on the analytical method reported in a previous study by Lemes et al. [22]. Separation was achieved using a 30-m HP5MS capillary column [(5%-phenyl)-methylpolysiloxane; Agilent J&W, Santa Clara, CA, USA] with an inner diameter of 0.25 mm and a film thickness of 0.25 µm. The oven temperature was initially held at 50°C for 1 minute, then increased at a rate of 6°C/minute to 300°C. The injection volume was 1 µL, with a split ratio of 20:1, and the injector temperature was set at 300 °C.

Oil samples were diluted with methanol to a concentration of 500 µg/mL, sonicated for 15 minutes, and filtered through a 0.45-μm nylon membrane. A 1 mL aliquot was transferred into an autosampler vial (Agilent 7693A, Santa Clara, CA, USA). A calibration curve for D-limonene was prepared using standard solutions in methanol at concentrations ranging from 50 to 500 µg/mL. Quantification was performed based on the peak area using the calibration curve. The analyses were performed in triplicate.

### Preparation and quantitative analysis of *O. sanctum* L. ethanolic extract

Dried *O. sanctum* L. powder (300 g) was macerated with 1000 ml of 95% ethanol for 5 days with occasional shaking [28]. The mixture was then filtered through Whatman No. 1 filter paper, and the filtrate was concentrated using a rotary evaporator (Model R-300, Buchi, Chadderton, England). The concentrate was subsequently dried at 50°C in a water bath (Memmert WNB 22, Schwabach, Germany). The percentage yield of the crude extract was calculated using the following formula:


$$Yield\;(\%)\;=\;\left(\frac{Weight\;of\;dried\;extract}{Weight\;of\;dried\;plant\;material}\right)\times 100$$

Quantitative analysis of ursolic acid, the biomarker of *O. sanctum* L., was performed according to the United States Pharmacopeia [29], with some modifications and validated procedures in compliance with the ICH Q2(R1) guidelines [30]. The analysis employed an HPLC system (Waters Alliance e2695, Waters Corporation, Milford, MA, USA) equipped with a Waters 2489 UV/Vis Detector. Chromatographic separation was carried out using a Hypersil BDS C18 column (5 µm, 250 × 4.6 mm; Thermo Fisher Scientific Inc., Waltham, MA, USA) maintained at 30°C. The mobile phase consisted of acetonitrile and 2.5 mg/mL ammonium acetate in water (70:30, v/v), delivered at a flow rate of 1.5 mL/minute. Detection was performed at 205 nm with an injection volume of 20 µL.

OSE samples were prepared by dissolving the extract in methanol to a final concentration of 50 mg/mL. A standard calibration curve was constructed using ursolic acid standard solutions prepared at concentrations ranging from 0.05 to 0.30 mg/mL. Before HPLC injection, all solutions were sonicated for 15 minutes and filtered through a 0.45-µm nylon filter. Quantification was based on the peak area comparison with the ursolic acid calibration curve. The analyses were performed in triplicate.

### Determination of antimicrobial activity

The antimicrobial activities of Lime TH, Lime SF, and OSE were evaluated against oral pathogens, including *L. acidophilus* (ATCC 4356), *S. mutans* (ATCC 25175), *P. gingivalis* (ATCC 33277), *A. actinomycetemcomitans* (ATCC 29522), and *C. albicans* (ATCC 10231). All microbial strains were purchased from the American Type Culture Collection (ATCC, Manassas, VA, USA).

The minimum inhibitory concentration (MIC), the minimum bactericidal concentration (MBC), and the minimum fungicidal concentration (MFC) were determined by a microbroth dilution assay following the previously described method of Balouiri et al. [31] and Clinical and Laboratory Standards Institute (CLSI) guidelines [32–34], with slight modifications. The experiment protocols for the antimicrobial testing were reviewed and approved by the Naresuan University Institutional Biosafety Committee, Thailand (NUIBC MI 67-03-14, Certification no. 67-27).

#### Minimum inhibitory concentration (MIC).

The stock solutions of Lime TH, Lime SF, and OSE were prepared by dissolving each agent in its corresponding growth medium (Table 1) containing 2% DMSO and 2% Tween 80 to obtain a final concentration of 200 mg/mL. The stock solutions (200 µL) of Lime TH, Lime SF, or OSE crude extract were added into the wells of a 96-well microplate, followed by two-fold serial dilutions with the respective growth medium.

**Table 1:** Growth mediums and incubated conditions of oral pathogens.

Oral pathogens	Growth mediums	Incubated conditions
*L. acidophilus*	Lactobacillus MRS Broth/Agar	35 ± 2°C for 48 hours in conditions with 5–10% CO_2_
*S. mutans*	Brain Heart Infusion Broth/Agar	35 ± 2 °C for 24 hours in conditions with 5–10% CO_2_
*P. gingivalis*	Tryptic soy Broth/Agar supplemented with yeast extract (1 mg/mL), hemin (5 μg/mL) and vitamin K1 (1 μg/mL). Growth mediums of *P. gingivalis* were preincubated for 1 day before use.	35 ± 2 °C for 72 hours in broth and for 5–7 days on agar plates under anaerobic conditions
*A. actinomycetemcomitans*	Brain Heart Infusion Broth/Agar	35 ± 2 °C for 48 hours in condition with 5–10% CO_2_
*C. albicans*	Sabouraud Dextrose Broth/Agar	35 ± 2 °C for 24 hours in aerobic conditions

Microbial suspensions were prepared by adjusting their turbidity to match a 0.5 McFarland standard and subsequently diluted 1:100 in growth medium to achieve a concentration of 1–2 × 10⁶ CFU/mL for bacterial strains and 1–2 × 10⁴ CFU/mL for fungal strains. Then, 100 µL of the microbial suspension was inoculated into each well, resulting in a final inoculum concentration of 0.5–1 × 10^6^ CFU/mL for bacteria and 0.5–1 × 10^4^ CFU/mL for fungi. The microplates were incubated under the specific conditions described in Table 1.

The MIC was defined as the lowest concentration of each tested sample that visibly inhibited the growth of oral pathogens. It was determined by identifying the wells that remained clear (non-turbid) compared to the growth observed in the control wells (i.e., growth control). The negative control consisted of growth medium containing 2% DMSO and 2% Tween 80 with oral pathogens, confirming that the solvent system itself had no inhibitory or toxic effects. The positive control was 0.12% w/w chlorhexidine gluconate solution.

#### Minimum bactericidal concentration (MBC) or minimum fungicidal concentration (MFC) determination.

The MBC or MFC was defined as the lowest concentration of the tested samples that resulted in complete microbial killing. To determine MBC or MFC values, 10 µL aliquots were taken from wells showing no visible growth (non-turbid) and inoculated onto the appropriate agar medium, as specified in Table 1. The plates were incubated under conditions suited to each pathogen, and colony formation was assessed after incubation.

#### Time-kill assay.

Time-kill assay performed using the method described by Balouiri et al. [31], with some modifications. The concentrations used in the time-kill assay were based on the MIC values determined previously and included 1 × MIC, MBC, and optionally 2 × MIC or 3 × MIC. Each sample was prepared at double strength by dissolving it into the appropriate growth medium with 2% DMSO and 2% Tween 80 to ensure solubility. A 100 µL aliquot of each sample was added to a well, followed by 100 µL of oral pathogen suspension (1–2 × 10^6^ CFU/mL), and the contents were thoroughly mixed. Positive and negative controls were included, consistent with the MIC assay.

The microplate was incubated under the appropriate conditions specified in Table 1. At intervals of 0, 2, 4, 6, and 24 hours, and additionally at 48 and 72 hours for the *P. gingivalis*, a 10 µL aliquot was collected from each well. The test samples were diluted 1:10 (sample well) or 1:100 (control) in growth medium. Then, 10 µL of the diluted sample was dropped in triplicate onto the appropriate agar, as indicated in Table 1. Following incubation, the number of colonies was counted to assess the viability of bacterial or fungal survival over time.

### Statistical analysis

All experiments were performed in triplicate. Results were expressed as mean ± standard deviation. Data analysis was performed using SPSS version 26.0 (IBM Corp., Armonk, NY, USA). Normality and homogeneity of variance were assessed using the Shapiro–Wilk test and Levene’s test, respectively. As the data were non-normally distributed and heterogeneous, non-parametric analysis was conducted using the Kruskal–Wallis test, followed by pairwise post hoc comparisons with Bonferroni correction. Statistical significance was defined as *p* < 0.05.

## Results

### Chemical compositions of *Citrus aurantifolia* peel oils

**Fig 1:**
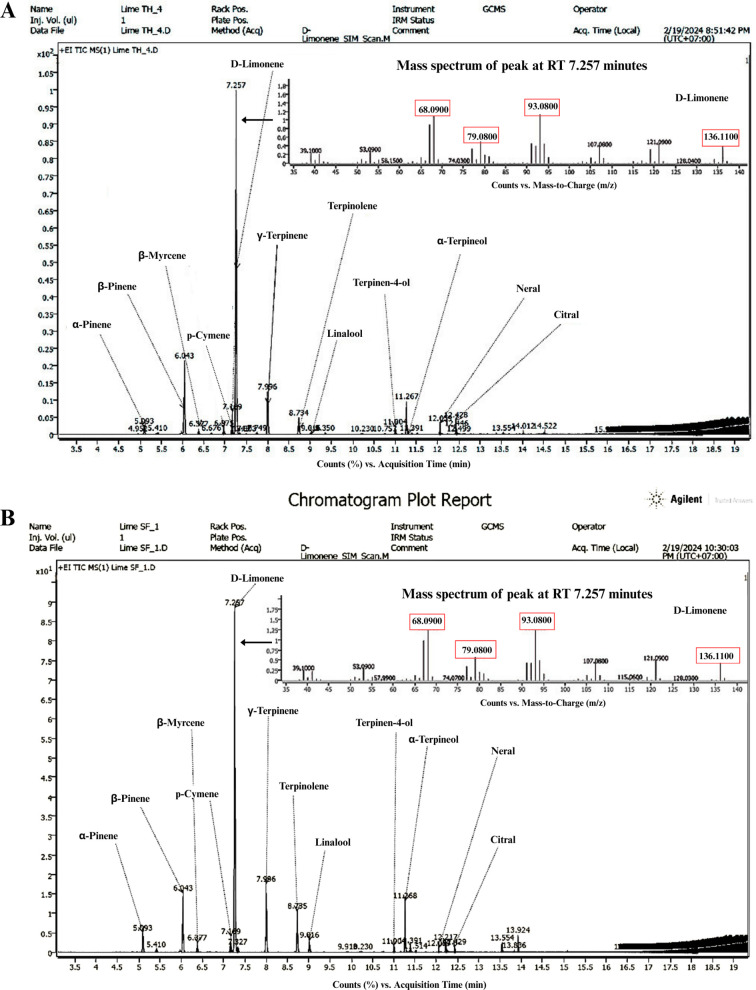
GC-MS chromatograms of *Citrus aurantifolia* peel oils. Chromatograms of peel oils obtained from **(A)** Thai lime (Lime TH) and **(B)** South African lime (Lime SF), showing D-limonene as the predominant component at a retention time of 7.25 minutes with characteristic ions at *m/z* 68, 79, 93, and 136. Twelve key volatile components were identified, including α-pinene, β-pinene, β-myrcene, p-cymene, D-limonene, γ-terpinene, terpinolene, linalool, terpinen-4-ol, α-terpineol, neral, and citral.

The chemical profiles of *C. aurantifolia* peel oils from Thai (Lime TH) and South African (Lime SF) sources are shown in Fig 1 and Table 2. GC-MS analysis revealed that both peel oils were predominantly composed of D-limonene, followed by β-pinene and γ-terpinene. The D-limonene peak was observed at a retention time of 7.25 minutes. The relative peak area, expressed as the percentage of a specific peak area divided by the total chromatographic peak area, is presented in Table 2.

**Table 2:** Major chemical constituents of *Citrus aurantifolia* peel oils. Comparison of retention time and relative peak area of key components, and match score in Lime TH and Lime SF based on GC-MS analysis.

No.	Components	Retention time	Lime TH (n = 3)			Lime SF (n = 3)		
			Peak area	Peak area (%)	Match Score	Peak area	Peak area (%)	Match Score
1	α-Pinene	5.09	83,741.67 ± 1,018.42	1.28 ± 0.01	913.67 ± 23.03	162,510.09 ± 1,201.35	2.55 ± 0.02	923.00 ± 9.54
2	β-Pinene	6.04	652,075.31 ± 13,720.35	9.96 ± 0.07	928.33 ± 3.79	446,836.36 ± 5,764.58	7.00 ± 0.01	926.00 ± 3.61
3	β-Myrcene	6.38	37,427.97 ± 762.77	0.57 ± 0.01	904.67 ± 37.58	56,736.46 ± 1,124.61	0.89 ± 0.01	912.33 ± 3.51
4	p-Cymene	7.17	88,655.89 ± 1,444.05	1.35 ± 0.01	928.67 ± 6.03	48,020.22 ± 415.29	0.76 ± 0.02	916.67 ± 11.02
5	D-Limonene	7.26	3,247,705.93 ± 54,141.90	49.59 ± 0.15	948.67 ± 9.29	2,763,946.07 ± 29,382.24	43.32 ± 0.23	951.67 ± 8.62
6	γ-Terpinene	8.00	423,099.27 ± 8,883.03	6.46 ± 0.06	928.67 ± 11.72	559,194.35 ± 3,845.50	8.77 ± 0.08	929.67 ± 4.16
7	Terpinolene	8.73	162,110.49 ± 3,900.62	2.48 ± 0.02	931.00 ± 14.42	340,830.66 ± 4,208.74	5.34 ± 0.03	936.00 ± 3.46
8	Linalool	9.02	4,873.95 ± 864.61	0.07 ± 0.01	637.33 ± 46.00	77,716.84 ± 661.94	1.22 ± 0.01	905.00 ± 3.46
9	Terpinen-4-ol	11.00	53,771.69 ± 1,417.52	0.82 ± 0.01	898.67 ± 19.35	37,734.35 ± 479.97	0.59 ± 0.01	839.67 ± 7.51
10	α-Terpineol	11.26	224,309.79 ± 6,124.75	3.43 ± 0.06	951.00 ± 8.66	315,263.91 ± 5,630.43	4.94 ± 0.07	947.00 ± 1.73
11	Neral	12.06	44,138.67 ± 1,334.92	0.67 ± 0.02	920.67 ± 12.42	8,192.64 ± 4,412.04	0.13 ± 0.07	832.00 ± 16.09
12	Citral	12.43	21,416.01 ± 641.50	0.33 ± 0.01	920.00 ± 13.00	12,116.53 ± 181.16	0.19 ± 0.00	838.33 ± 17.67

Quantitative analysis of D-limonene was performed using the selected ions for quantifier at *m/z* 68 and qualifier at *m/z* 79, 93, 136 in SIM mode. The concentration of D-limonene, calculated based on peak area and compared to the standard calibration curve (y = 2806.68x + 42373.97, R^2^ = 0.9984), showed that Lime TH contained 49.11 ± 0.76% w/w of D-limonene, while Lime SF contained 42.32 ± 0.60% w/w.

### Yield and ursolic acid content in *O. sanctum* ethanolic extract

**Fig 2:**
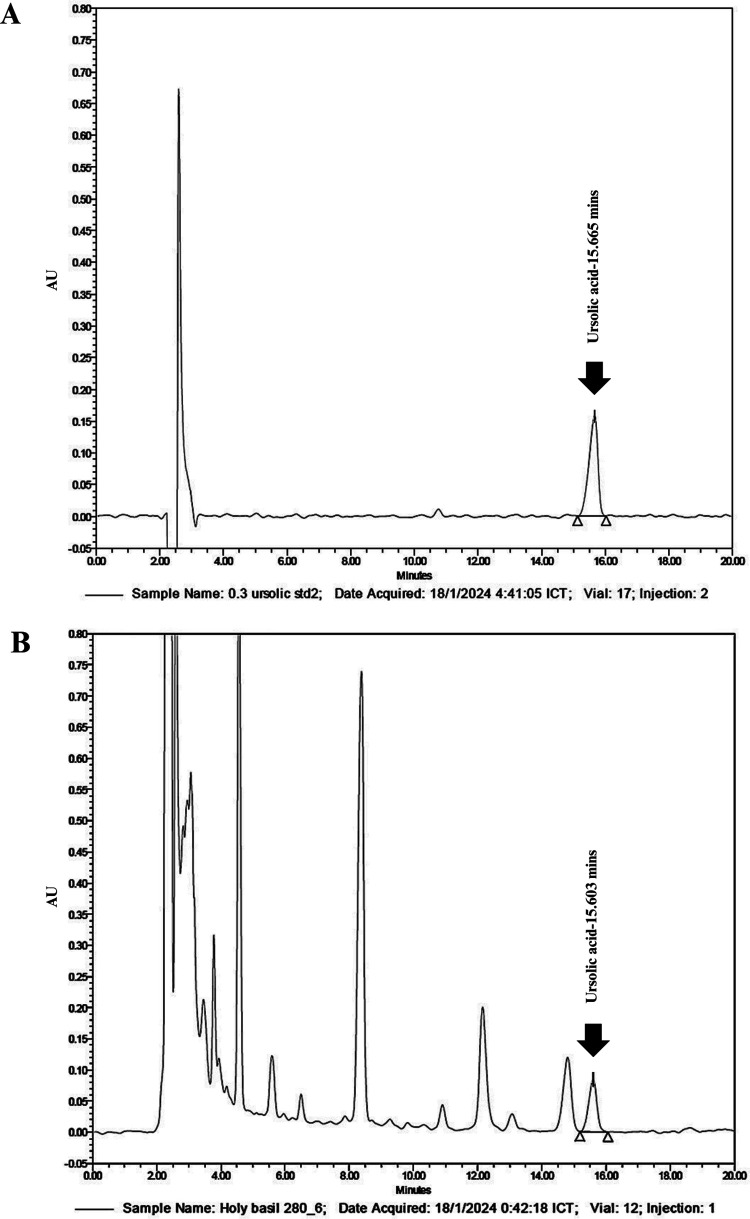
HPLC chromatograms of *Ocimum sanctum* ethanolic extract (OSE) and ursolic acid standard. (A) ursolic acid standard showing a sharp peak at 0.30 mg/mL and **(B)** OSE showing a complex phytochemical profile with multiple peaks at 50 mg/mL. Ursolic acid was identified in OSE with a retention time of 15.60 minutes.

The crude ethanolic extract of *O. sanctum*, obtained via maceration with 95% ethanol, yielded 4.59 ± 0.25% w/w. HPLC analysis of the extract showed a characteristic ursolic acid peak at a retention time of 15.60 minutes (Fig 2). The quantified ursolic acid content in the extract was 2.67 ± 0.07% w/w.

### Antimicrobial activity

#### Minimum inhibitory and bactericidal/fungicidal concentrations.

The MIC and MBC (or MFC) of Lime TH, Lime SF, and OSE against oral pathogens, including *L. acidophilus*, *S. mutans*, *P. gingivalis, A. actinomycetemcomitans*, and *C. albicans*, are presented in Table 3.

**Table 3:** MIC, MBC/MFC values, and MBC/MIC ratios of Lime TH, Lime SF, and OSE. Antimicrobial activity against five oral pathogens compared to 0.12% chlorhexidine gluconate (positive control).

Oral pathogens	Lime TH			Lime SF			OSE			0.12% CHX (Positive control)		
	MIC (mg/mL)	MBC (mg/mL)	MBC:MIC ratio	MIC (mg/mL)	MBC (mg/mL)	MBC:MIC ratio	MIC (mg/mL)	MBC (mg/mL)	MBC:MIC ratio	MIC (mg/mL)	MBC (mg/mL)	MBC:MIC ratio
*L. acidophilus*	12.50	25.00	2	12.50	50.00	4	50.0	>100.00	ND	1.20	1.20	1
*S. mutans*	1.56	12.50	8	3.13	25.00	8	12.50	100.00	8	1.20	1.20	1
*P. gingivalis*	0.20	0.78	4	0.39	1.56	4	0.05	0.20	4	1.20	1.20	1
*A. actinomycetemcomitans*	3.13	12.50	4	12.5	50.00	4	3.13	12.50	4	1.20	1.20	1
*C. albicans*	0.78	1.56	2	0.78	3.13	4	>100	>100.00	ND	1.20	1.20	1

All tests were performed in triplicate (n = 3), and all negative control wells (2% DMSO + 2% Tween 80 in growth medium) showed visible growth of all tested pathogens.

ND: Not determined.

Lime TH showed strong antimicrobial activity across all tested organisms, with MIC values ranging from 0.20 to 12.5 mg/mL and MBC/MFC values from 0.78 to 25.0 mg/mL. Lime SF exhibited slightly reduced potency, with MIC values ranging from 0.39 to 12.5 mg/mL and MBC/MFC values from 1.56 to 50.0 mg/mL.

OSE showed selective efficacy, particularly against *P. gingivalis* and *A. actinomycetemcomitans*, but was ineffective against *C. albicans* (MIC > 100 mg/mL). Chlorhexidine (0.12% w/w) served as the positive control, demonstrating uniform activity against all pathogens (MIC and MBC/MFC: 1.20 mg/mL). The negative control, comprising 2% DMSO and 2% Tween 80 in growth medium, showed uninhibited growth of all oral pathogens, confirming that the solvents did not exert antimicrobial effects at the tested concentrations.

#### Time-kill assay.

**Fig 3:**
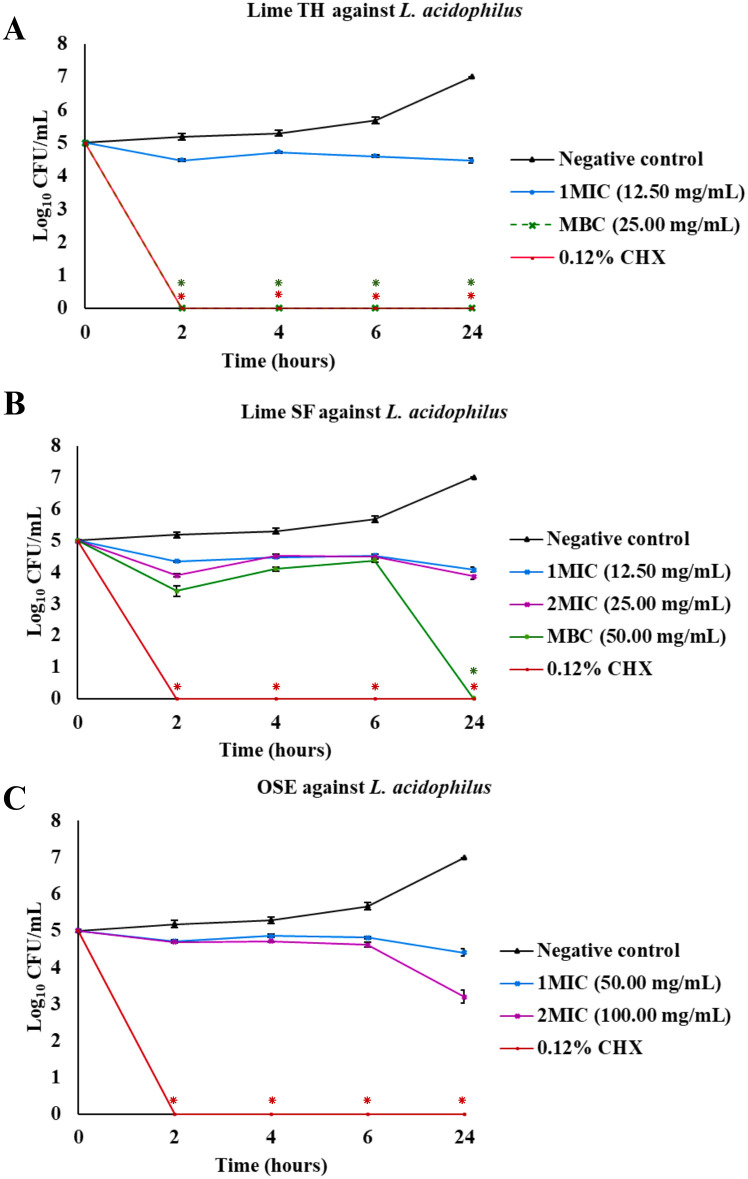
Time-kill curves showing the antimicrobial activity of test agents against *Lactobacillus acidophilus.* Each panel shows the reduction in log_10_ CFU/mL over time for treatments with Lime TH **(A)**, Lime SF **(B)**, and OSE **(C)**. Each data point represents the mean ± SD (n = 3). Asterisks indicate significant differences compared to the untreated control at corresponding time points (**p* *< 0.05).

**Fig 4:**
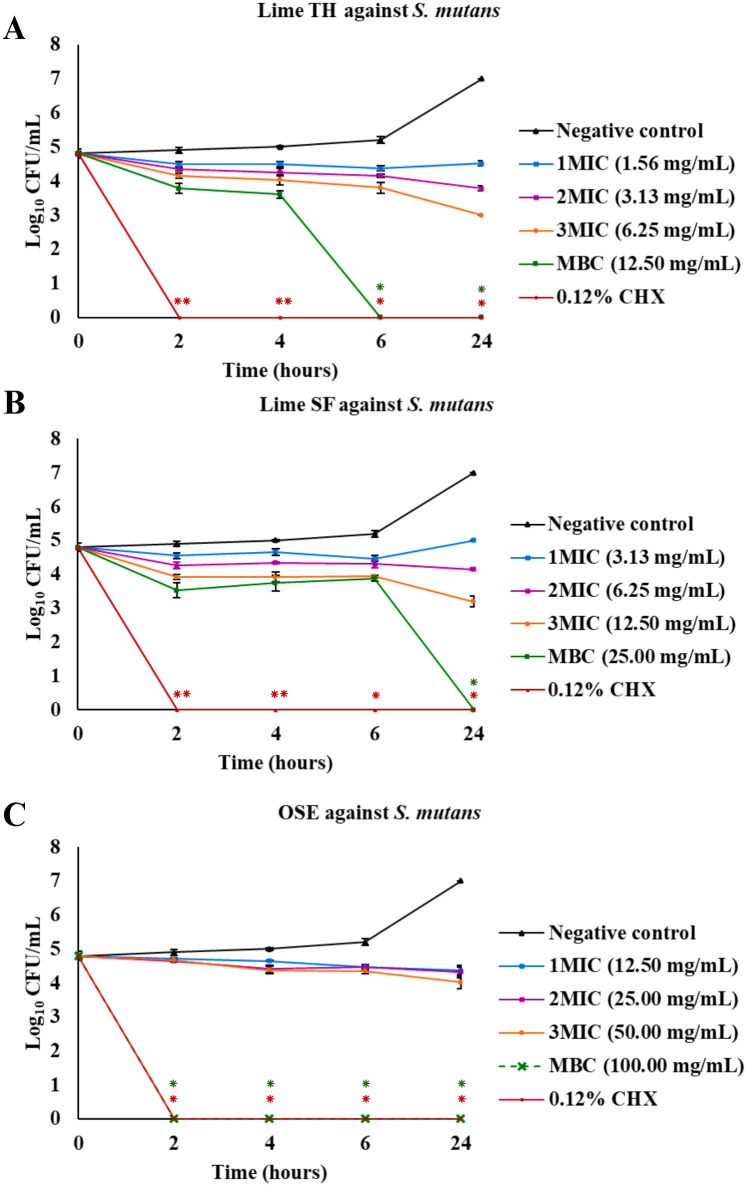
Time-kill curves showing the antimicrobial activity of test agents against *Streptococcus mutans.* Each panel shows the reduction in log_10_ CFU/mL over time for treatments with Lime TH **(A)**, Lime SF **(B)**, and OSE **(C)**. Each data point represents the mean ± SD (n = 3). Asterisks indicate significant differences compared to the untreated control at corresponding time points (*p < 0.05; **p < 0.01).

**Fig 5:**
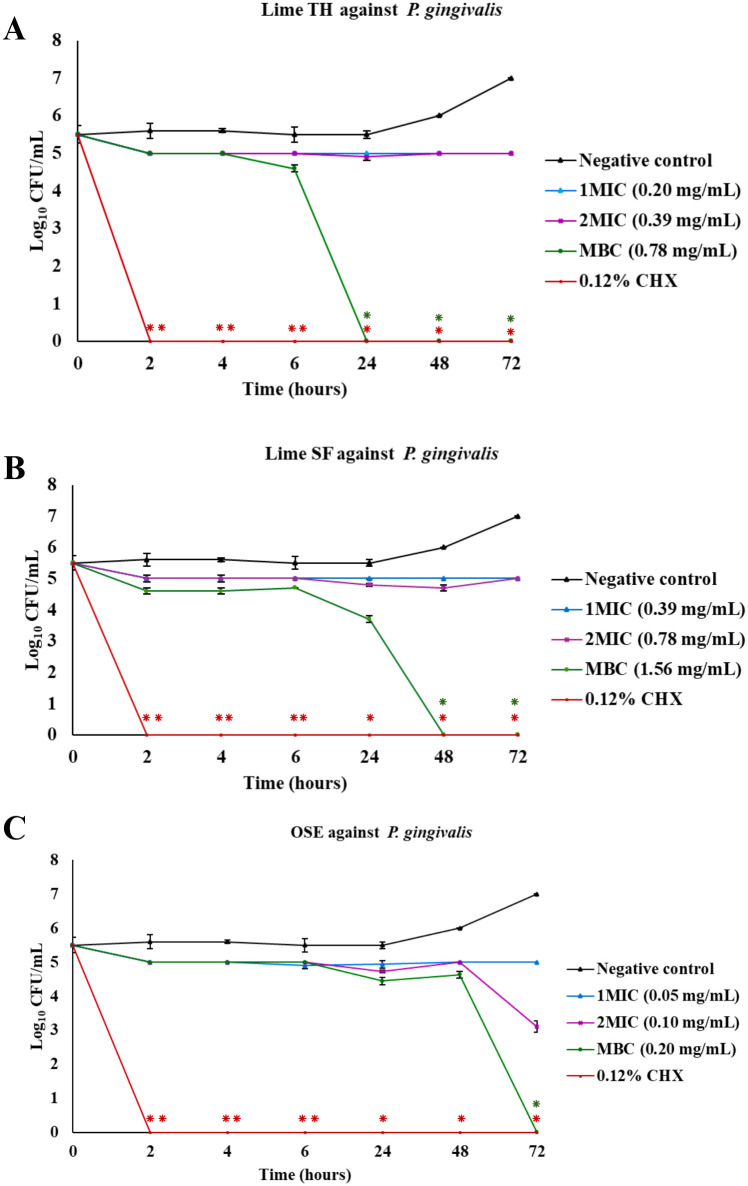
Time-kill curves showing the antimicrobial activity of test agents against *Porphyromonas gingivalis.* Each panel shows the reduction in log_10_ CFU/mL over time for treatments with Lime TH **(A)**, Lime SF **(B)**, and OSE **(C)**. Each data point represents the mean ± SD (n = 3). Asterisks indicate significant differences compared to the untreated control at corresponding time points (*p < 0.05; **p < 0.01).

**Fig 6:**
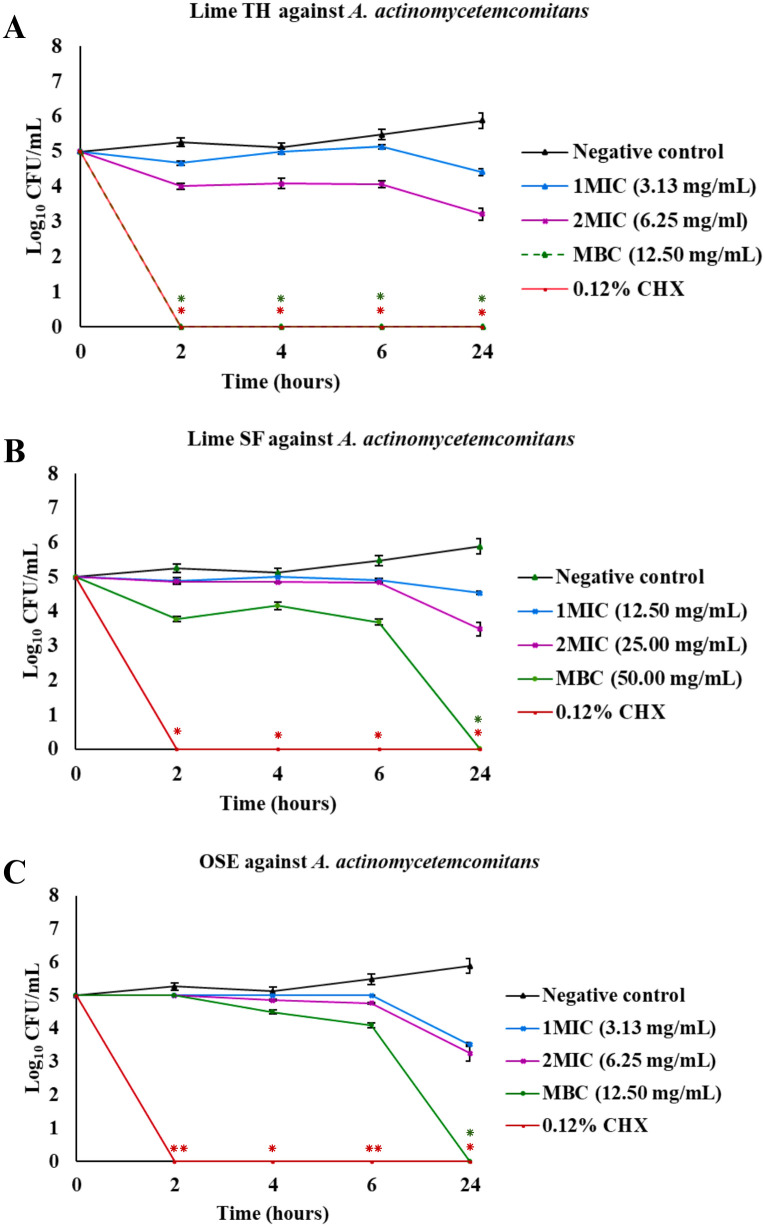
Time-kill curves showing the antimicrobial activity of test agents against *Aggregatibacter actinomycetemcomitans.* Each panel shows the reduction in log_10_ CFU/mL over time for treatments with Lime TH **(A)**, Lime SF **(B)**, and OSE **(C)**. Each data point represents the mean ± SD (n = 3). Asterisks indicate significant differences compared to the untreated control at corresponding time points (*p < 0.05; **p < 0.01).

**Fig 7:**
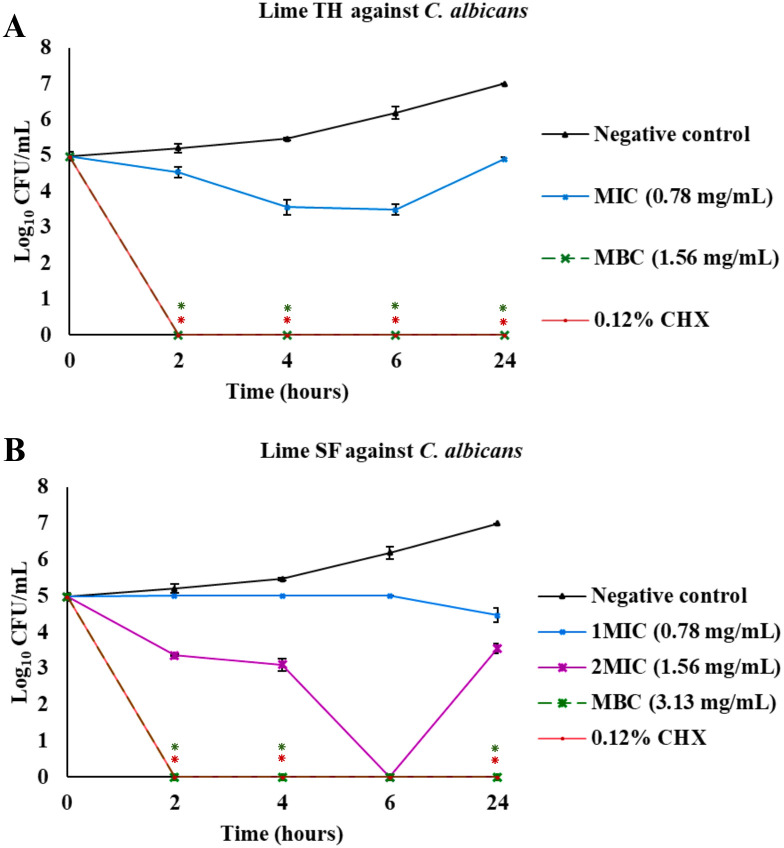
Time-kill curves showing the antimicrobial activity of test agents against *Candida albicans.* Each panel shows the reduction in log_10_ CFU/mL over time for treatments with Lime TH **(A)**, Lime SF **(B)**, and OSE **(C)**. Each data point represents the mean ± SD (n = 3). Asterisks indicate significant differences compared to the untreated control at corresponding time points (*p* < 0.05).

The results of the time-kill assays are presented as plots of log_10_ colony-forming units (CFU)/mL versus time (Figs 3–7, S1 Table). These plots illustrate the bactericidal or fungicidal activity of Lime TH, Lime SF, and OSE against the tested oral pathogens over time. These curves illustrate the time-dependent bactericidal and fungicidal effects of each treatment.

Lime TH demonstrated the most rapid kill of *L. acidophilus*, achieving complete inhibition within 2 hours at MBC concentration (Fig 3). Lime SF required up to 24 hours for similar effects. OSE showed delayed and incomplete killing. In *S. mutans*, Lime TH reduced viable counts completely by 6 hours, showing kinetics faster than Lime SF, which required 24 hours. OSE exhibited the most activity at MBC concentration, with complete bacterial clearance within 2 hours (Fig 4). Notably, all samples tested against *P. gingivalis* showed rapid and sustained bactericidal activity. Lime TH was particularly effective, eliminating the pathogen within 24 hours (Fig 5). For *A. actinomycetemcomitans*, Lime TH achieved bactericidal effects by 2 hours, while Lime SF and OSE showed slower action (Fig 6). In *C. albicans*, Lime TH and Lime SF reduced fungal viability in a time-dependent manner (Fig 7). According to the MIC/MFC data, OSE exhibited no fungicidal action.

## Discussion

*Citrus aurantifolia* (lime) and *Ocimum sanctum* (holy basil) are extensively cultivated in Thailand and are recognized for their potential as natural agents in oral healthcare. Previous studies have evaluated the antimicrobial activity of hydrodistilled *C. aurantifolia* peel essential oil primarily against *Streptococcus mutans* and *Candida albicans* [22,23]. However, some investigation employed the agar diffusion method, which can underestimate activity due to the high volatility of essential oils during incubation and their limited solubility in agar matrices. Furthermore, the antimicrobial activity of lime peel against other clinically relevant oral pathogens, including *Lactobacillus acidophilus*, *Porphyromonas gingivalis*, and *Aggregatibacter actinomycetemcomitans*, has been examined mainly using crude ethanolic extracts rather than hydrodistilled essential oils, despite the substantial differences in chemical composition between these preparations [24,25]. Similarly, the ethanolic extract of *O. sanctum* has been reported to exhibit activity only against *L. acidophilus*, *S. mutans*, and *A. actinomycetemcomitans* [26,27]. To address these gaps, the present study investigates both the chemical compositions and antimicrobial activities of *C. aurantifolia* peel oils sourced from Thailand (Lime TH) and South Africa (Lime SF, used as a commercially standardized reference) alongside the ethanolic extract of *O. sanctum* (OSE) against five clinically important oral pathogens.

Gas chromatography–mass spectrometry (GC-MS) analysis revealed D-limonene as the predominant compound in both lime oils, with higher content in Lime TH (49.11 ± 0.76% w/w) than in Lime SF (42.32 ± 0.60% w/w). High-performance liquid chromatography (HPLC) confirmed the presence of ursolic acid in OSE at 2.67 ± 0.07% w/w, which was slightly higher than previously reported in Brazilian *O. sanctum* extracts (2.02% w/w) [35]. These compositional differences may be attributed to geographical and environmental factors, including soil composition, climate, and harvesting conditions, which are known to influence phytochemical profiles [36].

The antimicrobial efficacy of three natural agents was evaluated against *L. acidophilus*, *S. mutans*, *P. gingivalis*, *A. actinomycetemcomitans*, and *C. albicans*. Based on MBC/MIC ratios, activities were classified as bactericidal (ratio ≤ 4) or bacteriostatic (ratio > 4) [37,38]. Additionally, these effects were confirmed by the time-kill assay. A reduction in oral pathogens of less than 3 log₁₀ was defined as bacteriostatic, while a reduction of ≥ 3 log₁₀ in total microbial count (CFU/mL), representing 99.9% elimination of the oral pathogens, was defined as bactericidal. Regrowth was defined as an increase of ≥ 2 log₁₀ in total microbial count (CFU/mL) after ≥ 6 hours [39,40].

*S. mutans* is considered the primary etiological agent of dental caries in humans [41]. While *S. mutans* initiates caries development, *L. acidophilus* may be more significant in the progression of existing caries lesions [42]. *L. acidophilus* is investigated as probiotics to inhibit *S. mutans*, reduce biofilm formation, and prevent caries [43,44]. In healthy individuals, *L. acidophilus* is typically absent or present in low numbers but increases markedly in active caries sites [45,46]. In this study, all tested agents exhibited stronger antimicrobial activity against *S. mutans* than against *L. acidophilus*. At sub-MBC concentrations, lime peel oils and OSE exhibited only bacteriostatic effects on *L. acidophilus*, supporting their potential application in microbiome-friendly oral care products designed to suppress pathogens without eliminating commensal species [47,48]. At MBC levels, these agents demonstrated bactericidal effects, which may be advantageous for therapeutic formulations intended for active caries management.

Among the tested agents, Lime TH demonstrated the strongest activity against *S. mutans* (MIC 1.56 mg/mL, MBC 12.50 mg/mL), while Lime SF required higher concentrations (MIC 3.13 mg/mL, MBC 25.00 mg/mL). The MBC/MIC ratios for both oils were > 4, indicating a bacteriostatic classification (Table 3). However, time–kill assays revealed that both oils achieved bactericidal effects at their respective MBCs: Lime TH produced a 3 log₁₀ reduction within 6 hours, while Lime SF required 24 hours (Fig 4 and S1 Table). These findings are consistent with previous reports of the anti-cariogenic activity of lime oil and D-limonene [22]. OSE showed lower potency against *S. mutans* (MIC 12.50 mg/mL; MBC 100.00 mg/mL; MBC/MIC = 8), suggesting a bacteriostatic effect. Nonetheless, at its MBC concentration, OSE achieved a 3 log₁₀ reduction within 2 hours.

The anti-cariogenic effects of lime peel oil are largely attributed to D-limonene [49–51]. As a Gram-positive bacterium, *S. mutans* possesses a thick, mesh-like peptidoglycan cell wall surrounding a single cytoplasmic membrane [52]. The rigid peptidoglycan network provides structural support and protects against osmotic lysis, while the underlying phospholipid bilayer maintains cellular integrity. D-limonene (~136 Da; log P = 5.41 [53]) is a low molecular weight, highly lipophilic monocyclic monoterpene hydrocarbon. Its structure activity relationship (SAR) is dominated by hydrophobic membrane partitioning. It can penetrate the cell wall and reach the cytoplasmic membrane, causing membrane fluidization, altering membrane potential, increased permeability, and leakage of intracellular contents; at high concentrations this can lead to cell lysis [51,54]. In addition, *S. mutans* adheres to tooth surfaces and produces extracellular polysaccharides (EPS) through glucosyltransferases (GTFs) activity. D-limonene impairs the adhesion and biofilm formation by down-regulating surface/virulence genes and interfering with EPS production, thereby reducing acid retention and enhancing susceptibility to environmental stress [51,55].

From a SAR perspective, ursolic acid (~456 Da; log P = 7.09 [56]) is also lipophilic but possesses a larger, more rigid triterpenoid structure than D-limonene, which may hinder its penetration into bacterial cells. While limonene primarily disrupts membranes, ursolic acid may exert its antimicrobial effects through interactions with bacterial surface proteins or enzymes. Notably, Liu et al. [57] demonstrated that ursolic acid inhibits cariogenic biofilm formation by directly binding to bacterial GTFs, thereby blocking EPS synthesis, reducing bacterial viability, and compromising biofilm structural integrity. Given that OSE is a crude ethanolic extract containing multiple bioactive constituents, the antimicrobial mechanism observed in this study likely results from a combination of actions, and the specific contribution of ursolic acid cannot be conclusively determined.

*P. gingivalis* and *A. actinomycetemcomitans* are key periodontal pathogens. *P. gingivalis* is more commonly associated with severe chronic periodontitis in both young and adult patients, while *A. actinomycetemcomitans* linked to aggressive periodontitis in younger individuals [58]. In this study, Lime TH showed the highest potency against *P. gingivalis* (MIC 0.20 mg/mL; MBC 0.78 mg/mL), followed by Lime SF (MIC 0.39 mg/mL; MBC 1.56 mg/mL). Both oils were bactericidal (MBC/MIC ≤ 4). In time–kill assays, Lime TH achieved a ≥ 3 log₁₀ reduction within 24 hours, while Lime SF required 48 hours (Fig 5; S1 Table). OSE inhibited *P. gingivalis* growth but required 72 hours at its MBC for bactericidal effects.

For *A. actinomycetemcomitans*, previous studies have reported antimicrobial activity of OSE at 5–10% w/w [59], but no prior data exist for lime peel oils. Here, Lime TH and OSE both showed MIC and MBC values of 3.13 mg/mL and 12.50 mg/mL, respectively, while Lime SF was less potent (MIC 12.50 mg/mL; MBC 50.00 mg/mL). Time–kill assays indicated that at MBC levels, Lime TH achieved bactericidal activity within 2 hours, compared to 24 hours for both OSE and Lime SF (Fig 6).

Both *P. gingivalis* and *A. actinomycetemcomitans* are Gram-negative bacteria with a tripartite cell envelope comprising an outer membrane, a thin peptidoglycan layer, and an inner cytoplasmic membrane [52]. These pathogens cause periodontitis through direct infection and by delivering virulence factors into host cells, often via outer membrane vesicles (OMVs), which contribute to tissue destruction and inflammation [60]. D-limonene, a small lipophilic monoterpene, partitions into the lipid-rich outer membrane and OMVs, disrupting membrane integrity and permeability. Such disruption may impair the stability and release of key virulence enzymes, including peptidyl arginine deiminase (PPAD) from *P. gingivalis*, which catalyzes protein citrullination and promotes tissue-destructive inflammation, and dispersin B from *A. actinomycetemcomitans*, which degrades poly-N-acetylglucosamine (PGA) biofilm matrix [61,62].

The molecular mechanisms of ursolic acid against these periodontal pathogens are less well characterized. Ursolic acid exhibits general antibacterial and antibiofilm activity against Gram-negative bacteria, likely due to its high lipophilicity, enabling insertion into lipid-rich OMVs and bacterial membranes, leading to leakage of vesicle contents and destabilization of biofilms [63]. From a SAR perspective, ursolic acid’s higher molecular weight and rigid triterpenoid skeleton limit rapid membrane penetration, but its high log P allows strong insertion into hydrophobic domains once access is gained. This may destabilize OMVs and compromise biofilm matrices, explaining its observed bactericidal effects against Gram-negative species despite slower killing kinetics.

*C. albicans*, the predominant cause of oral candidiasis, was most susceptible to Lime TH (MFC 1.56 mg/mL) compared with Lime SF (MFC 3.13 mg/mL), though both achieved > 99.9% killing within 2 hours (Fig 7; S1 Table). OSE exhibited no antifungal activity up to 100.00 mg/mL.

The *C. albicans* cell wall contains an outer glycoprotein–mannan layer and an inner structural layer of chitin and glucans [64]. Limonene exposure activates cell wall integrity signaling genes (e.g., *ROM1*, *RLM1*, *PIR3*, *CTT1*, *YGP1*, *MLP1*, *PST1*, *CWP1*), indicating stress and weakening of the cell wall [65]. This is followed by plasma membrane disruption, increased permeability, and ion leakage, triggering ROS accumulation, oxidative stress, apoptosis, and DNA damage [66]. In SAR terms, limonene’s low molecular weight and high hydrophobicity facilitate rapid penetration and membrane destabilization, explaining the fast fungicidal action observed. Ursolic acid, in contrast, penetrates more slowly due to steric bulk, resulting in delayed or absent antifungal activity under the tested conditions.

This study is the first to compare *C. aurantifolia* peel oils from different geographical origins alongside *O. sanctum* ethanolic extract against a clinically relevant spectrum of oral pathogens, integrating MIC, MBC/MFC, and time–kill assays for robust classification of bacteriostatic/bactericidal effects. The SAR-based discussion links phytochemical composition to pathogen-specific mechanisms, supporting rational formulation design. Limitations include the *in vitro* nature of the assays, lack of cytotoxicity testing on human oral cells, and absence of biofilm or multispecies models that better simulate oral environments. Furthermore, the crude nature of OSE complicates attribution of activity to specific compounds. Future studies should evaluate safety margins, biofilm activity, and stability within realistic oral care formulations.

## Conclusion

This study provides detailed insights into the chemical composition and antimicrobial potential of *Citrus aurantifolia* peel oils from Thailand and South Africa and *Ocimum sanctum* ethanolic extract. By quantifying key bioactive compounds (D-limonene and ursolic acid) and evaluating their antimicrobial activity against major oral pathogens using MIC, MBC/MFC, and time-kill assays, the findings demonstrate the potential of these natural agents for combating oral pathogens associated with dental caries, periodontal diseases, and fungal infections. Lime TH exhibited the strongest and broad-spectrum activity, supporting its potential for incorporation into oral care formulations such as mouth rinses, oral gels, or dentifrices. However, as the experiments were conducted under *in vitro* conditions, physiological factors such as salivary enzymes, pH fluctuations, biofilm complexity, and oral mucosal irritation were not evaluated. Therefore, further studies should include *in vivo* experiments, irritation testing using 3D oral mucosal models, and formulation-based investigations to confirm clinical efficacy, safety, and long-term stability.

## Supporting information

S1 Table Log_10_ reduction in time-kill assay of the tested natural agent. (DOCX)

## References

[1] World Health Organization. WHO global oral health status report: Towards universal health coverage for oral health by 2030 [Internet]. Geneva: World Health Organization; 2023 [cited 2025 Jul 23]. Available from: https://www.who.int

[2] Vaou N, Stavropoulou E, Voidarou C, Tsigalou C, Bezirtzoglou E. Towards Advances in Medicinal Plant Antimicrobial Activity: A Review Study on Challenges and Future Perspectives. Microorganisms. 2021;9(10):2041. doi: 10.3390/microorganisms9102041 34683362 PMC8541629

[3] Laudenbach JM, Kumar SS. Common Dental and Periodontal Diseases. Dermatol Clin. 2020;38(4):413–20. doi: 10.1016/j.det.2020.05.002 32892850

[4] Loesche WJ. Microbiology of dental decay and periodontal disease. In: Baron S, editor. Medical microbiology [Internet]. 4th ed. Galveston (TX): University of Texas Medical Branch; 1996 [cited 2025 Jul 23]. Available from: https://www.ncbi.nlm.nih.gov/books/NBK8259/21413316

[5] Baker JL, Edlund A. Exploiting the Oral Microbiome to Prevent Tooth Decay: Has Evolution Already Provided the Best Tools? Front Microbiol. 2019;9:3323. doi: 10.3389/fmicb.2018.03323 30687294 PMC6338091

[6] Tahmourespour A, Salehi R, Kasra Kermanshahi R. Lactobacillus Acidophilus-Derived Biosurfactant Effect on GTFB and GTFC Expression Level in Streptococcus Mutans Biofilm Cells. Braz J Microbiol. 2011;42(1):330–9. doi: 10.1590/S1517-83822011000100042 24031639 PMC3768947

[7] Zhao J, Le K, Feng X, Ma L. Antagonistic effects of Lactobacillus acidophilus and Bifidobacterium adolescents on periodontalpathogens in vitro. Shanghai Kou Qiang Yi Xue. 2011;20(4):364–7. 21909593

[8] Ishikawa KH, Bueno MR, Kawamoto D, Simionato MRL, Mayer MPA. Lactobacilli postbiotics reduce biofilm formation and alter transcription of virulence genes of Aggregatibacter actinomycetemcomitans. Mol Oral Microbiol. 2021;36(1):92–102. doi: 10.1111/omi.12330 33372378

[9] Jaffar N, Ishikawa Y, Mizuno K, Okinaga T, Maeda T. Mature Biofilm Degradation by Potential Probiotics: Aggregatibacter actinomycetemcomitans versus Lactobacillus spp. PLoS One. 2016;11(7):e0159466. doi: 10.1371/journal.pone.0159466 27438340 PMC4954673

[10] Ferreira MC, Dias-Pereira AC, Branco-de-Almeida LS, Martins CC, Paiva SM. Impact of periodontal disease on quality of life: a systematic review. J Periodontal Res. 2017;52(4):651–65. doi: 10.1111/jre.12436 28177120

[11] Loesche WJ, Grossman NS. Periodontal disease as a specific, albeit chronic, infection: diagnosis and treatment. Clin Microbiol Rev. 2001;14(4):727–52, table of contents. doi: 10.1128/CMR.14.4.727-752.2001 11585783 PMC89001

[12] Könönen E, Müller H-P. Microbiology of aggressive periodontitis. Periodontol 2000. 2014;65(1):46–78. doi: 10.1111/prd.12016 24738586

[13] Marcano R, Rojo MÁ, Cordoba-Diaz D, Garrosa M. Pathological and Therapeutic Approach to Endotoxin-Secreting Bacteria Involved in Periodontal Disease. Toxins (Basel). 2021;13(8):533. doi: 10.3390/toxins13080533 34437404 PMC8402370

[14] Coronado-Castellote L, Jiménez-Soriano Y. Clinical and microbiological diagnosis of oral candidiasis. J Clin Exp Dent. 2013;5(5):e279-86. doi: 10.4317/jced.51242 24455095 PMC3892259

[15] Mansur EKM. Primary Prevention of Dental Caries: An Overview. Int J Clin Prev Dent. 2020;16(4):143–8. doi: 10.15236/ijcpd.2020.16.4.143

[16] Ghaemi Z, Noshadi M. Evaluation of fluoride exposure using disability-adjusted life years and health risk assessment in south-western Iran: A novel Monte Carlo simulation. Ecotoxicol Environ Saf. 2024;282:116705. doi: 10.1016/j.ecoenv.2024.116705 39003868

[17] DenBesten P, Li W. Chronic fluoride toxicity: dental fluorosis. Monogr Oral Sci. 2011;22:81–96. doi: 10.1159/000327028 21701193 PMC3433161

[18] Nassar Y, Brizuela M. The role of fluoride on caries prevention. In: StatPearls [Internet]. Treasure Island (FL): StatPearls Publishing; 2023 Mar 19 [cited 2025 May 16]. Available from: https://www.ncbi.nlm.nih.gov/books/NBK587342/36508516

[19] Ministry of Public Health. Notification of the Ministry of Public Health: specification of names, quantities, and conditions of substances permitted for use in cosmetic products (No. 3), B.E. 2019. R Thai Gov Gaz. 2020 Feb 18;137(Special Section 37 Ng).

[20] Sarembe S, Ufer C, Kiesow A, Limeback H, Meyer F, Fuhrmann I, et al. Influence of the Amount of Toothpaste on Cleaning Efficacy: An In Vitro Study. Eur J Dent. 2023;17(2):497–503. doi: 10.1055/s-0042-1747953 35785824 PMC10329550

[21] Sajjan P, Laxminarayan N, Kar PP, Sajjanar M. Chlorhexidine as an antimicrobial agent in dentistry: a review. Oral Health Dent Manag. 2016;15(2):93–100.

[22] Lemes RS, Alves CCF, Estevam EBB, Santiago MB, Martins CHG, Santos TCLD, et al. Chemical composition and antibacterial activity of essential oils from Citrus aurantifolia leaves and fruit peel against oral pathogenic bacteria. An Acad Bras Cienc. 2018;90(2):1285–92. doi: 10.1590/0001-3765201820170847 29898096

[23] Mohammed IO, Alrasheid AA, Hussein Ayoub SM. GC-MS Analysis and Study of the Antimicrobial Activity of Citrus paradisi, Citrus aurantifolia, and Citrus sinensis Peel Essential Oils as Hand Sanitizer. Int J Microbiol. 2024;2024:4957712. doi: 10.1155/2024/4957712 38204865 PMC10776194

[24] Nugraha PY, Astuti ESY, Saraswati IAPMC. Lime peel extract (Citrus aurantifolia) inhibit the growth of bacteria Lactobacillus acidophilus in childhood dental caries. Int J Dent Sci. 2023;1(1):14–28.

[25] Pasaribu F, Ervina I, Suryanto D. The effectiveness antimicrobial activity test of citrus peel extract on some periodontal pathogenic bacteria (in vitro). Int J Appl Dent Sci. 2018;4(3):146–50.

[26] Gadiyar A, Ankola AV, Rajpurohit L. Evaluation of the antimicrobial activity of Ocimum sanctum L. (Tulsi) extract against Streptococcus mutans and Lactobacillus acidophilus—an in vitro study. Int J Health Sci Res. 2017;7(4):224–8.

[27] Eswar P, Devaraj CG, Agarwal P. Anti-microbial Activity of Tulsi {Ocimum Sanctum (Linn.)} Extract on a Periodontal Pathogen in Human Dental Plaque: An Invitro Study. J Clin Diagn Res. 2016;10(3):ZC53-6. doi: 10.7860/JCDR/2016/16214.7468 27135002 PMC4843387

[28] Chanthaboury M, Choonharuangdej S, Shrestha B, Srithavaj T. Antimicrobial Properties of Ocimum Species: An In Vitro Study. J Int Soc Prev Community Dent. 2022;12(6):596–602. doi: 10.4103/jispcd.JISPCD_155_22 36777016 PMC9912833

[29] United States Pharmacopeia (USP). Powdered Holy Basil Leaf Extract [Internet]. Rockville (MD): United States Pharmacopeial Convention; 2020 May 1 [cited 2025 Jul 23]. Available from: https://online.uspnf.com/uspnf/document/GUID-7C663AD7-5D4E-4DB4-AD28-083A93F80F45

[30] International Conference on Harmonisation (ICH). Validation of analytical procedures: text and methodology Q2(R1). ICH Harmonised Tripartite Guideline. 2005;1(20):05.

[31] Balouiri M, Sadiki M, Ibnsouda SK. Methods for in vitro evaluating antimicrobial activity: A review. J Pharm Anal. 2016;6(2):71–9. doi: 10.1016/j.jpha.2015.11.005 29403965 PMC5762448

[32] Clinical and Laboratory Standards Institute (CLSI). Method for broth dilution antifungal susceptibility testing of yeasts. Approved standard M27-A02. Wayne (PA): CLSI; 2002.

[33] Clinical and Laboratory Standards Institute (CLSI). Methods for antimicrobial susceptibility testing of anaerobic bacteria. Approved standard M11-A07. Wayne (PA): CLSI; 2007.

[34] Clinical and Laboratory Standards Institute (CLSI). Methods for dilution antimicrobial susceptibility tests for bacteria that grow aerobically. Approved standard M07-A09. Wayne (PA): CLSI; 2012.

[35] Silva MGV, Vieira IGP, Mendes FNP, Albuquerque IL, dos Santos RN, Silva FO, et al. Variation of ursolic acid content in eight Ocimum species from northeastern Brazil. Molecules. 2008;13(10):2482–7. doi: 10.3390/molecules13102482 18923339 PMC6245192

[36] Masotti V, Juteau F, Bessière JM, Viano J. Seasonal and phenological variations of the essential oil from the narrow endemic species Artemisia molinieri and its biological activities. J Agric Food Chem. 2003;51(24):7115–21. doi: 10.1021/jf034621y 14611181

[37] Mujawah AAH, Abdallah EM, Alshoumar SA, Alfarraj MI, Alajel SMI, Alharbi AL, et al. GC-MS and in vitro antibacterial potential of Cinnamomum camphora essential oil against some clinical antibiotic-resistant bacterial isolates. Eur Rev Med Pharmacol Sci. 2022;26(15):5372–9. doi: 10.26355/eurrev_202208_29404 35993631

[38] Stefanovic O, Stankovic MS, Comic L. In vitro antibacterial efficacy of Clinopodium vulgare L. extracts and their synergistic interaction with antibiotics. J Med Plant Res. 2011;5(17):4074–9.

[39] Seephonkai P, Sedlak S, Wongpakam K, Sangdee K, Sangdee A. Time-kill kinetics and mechanism of action of Caesalpinia sappan L. and Ochna integerrima (Lour.) Merr. water extracts against pathogenic bacteria. J Pharm Pharmacol Res. 2021;9(6):813–23.

[40] McMurray RL, Ball MEE, Tunney MM, Corcionivoschi N, Situ C. Antibacterial Activity of Four Plant Extracts Extracted from Traditional Chinese Medicinal Plants against Listeria monocytogenes, Escherichia coli, and Salmonella enterica subsp. enterica serovar Enteritidis. Microorganisms. 2020;8(6):962. doi: 10.3390/microorganisms8060962 32604894 PMC7355567

[41] Hamada S, Slade HD. Biology, immunology, and cariogenicity of Streptococcus mutans. Microbiol Rev. 1980;44(2):331–84. doi: 10.1128/mr.44.2.331-384.1980 6446023 PMC373181

[42] Fu D, Shu X, Yao L, Zhou G, Ji M, Liao G, et al. Unveiling the dual nature of Lactobacillus: from cariogenic threat to probiotic protector-a critical review with bibliometric analysis. Front Oral Health. 2025;6:1535233. doi: 10.3389/froh.2025.1535233 39959355 PMC11825810

[43] Wasfi R, Abd El-Rahman OA, Zafer MM, Ashour HM. Probiotic Lactobacillus sp. inhibit growth, biofilm formation and gene expression of caries-inducing Streptococcus mutans. J Cell Mol Med. 2018;22(3):1972–83. doi: 10.1111/jcmm.13496 29316223 PMC5824418

[44] Ahmed A, Dachang W, Lei Z, Jianjun L, Juanjuan Q, Yi X. Effect of Lactobacillus species on Streptococcus mutans biofilm formation. Pak J Pharm Sci. 2014;27(5 Spec no):1523–8. 25176247

[45] Caufield PW, Schön CN, Saraithong P, Li Y, Argimón S. Oral Lactobacilli and Dental Caries: A Model for Niche Adaptation in Humans. J Dent Res. 2015;94(9 Suppl):110S-8S. doi: 10.1177/0022034515576052 25758458 PMC4547204

[46] Nunpan S, Suwannachart C, Wayakanon K. The inhibition of dental caries pathogen by using prebiotic and probiotic combination. J Dent Assoc Thail. 2017;67:31–8.

[47] El Enshasy HA, Niazy MA, Kamh R, Heng LH, Salaie RN. Recent development in oral hygiene products: from product development to market. In: Natural conservative dentistry: an alternative approach to solve restorative problems. Sharjah: Bentham Science Publishers; 2024. p. 239–53.

[48] Rajasekaran JJ, Krishnamurthy HK, Bosco J, Jayaraman V, Krishna K, Wang T, et al. Oral Microbiome: A Review of Its Impact on Oral and Systemic Health. Microorganisms. 2024;12(9):1797. doi: 10.3390/microorganisms12091797 39338471 PMC11434369

[49] Burt S. Essential oils: their antibacterial properties and potential applications in foods--a review. Int J Food Microbiol. 2004;94(3):223–53. doi: 10.1016/j.ijfoodmicro.2004.03.022 15246235

[50] Chouhan S, Sharma K, Guleria S. Antimicrobial Activity of Some Essential Oils-Present Status and Future Perspectives. Medicines (Basel). 2017;4(3):58. doi: 10.3390/medicines4030058 28930272 PMC5622393

[51] Lin H, Li Z, Sun Y, Zhang Y, Wang S, Zhang Q, et al. D-Limonene: Promising and Sustainable Natural Bioactive Compound. Appl Sci. 2024;14(11):4605. doi: 10.3390/app14114605

[52] Sharma P, Vaiwala R, Gopinath AK, Chockalingam R, Ayappa KG. Structure of the Bacterial Cell Envelope and Interactions with Antimicrobials: Insights from Molecular Dynamics Simulations. Langmuir. 2024;40(15):7791–811. doi: 10.1021/acs.langmuir.3c03474 38451026

[53] Schroder V, Radu N, Cornea PC, Coman OA, Pirvu LC, Mohammed MSO, et al. Studies Regarding the Antimicrobial Behavior of Clotrimazole and Limonene. Antibiotics (Basel). 2022;11(12):1816. doi: 10.3390/antibiotics11121816 36551473 PMC9774930

[54] Espina L, Gelaw TK, de Lamo-Castellví S, Pagán R, García-Gonzalo D. Mechanism of bacterial inactivation by (+)-limonene and its potential use in food preservation combined processes. PLoS One. 2013;8(2):e56769. doi: 10.1371/journal.pone.0056769 23424676 PMC3570463

[55] Subramenium GA, Vijayakumar K, Pandian SK. Limonene inhibits streptococcal biofilm formation by targeting surface-associated virulence factors. J Med Microbiol. 2015;64(8):879–90. doi: 10.1099/jmm.0.000105 26294065

[56] Mendie LE, Hemalatha S. Molecular Docking of Phytochemicals Targeting GFRs as Therapeutic Sites for Cancer: an In Silico Study. Appl Biochem Biotechnol. 2022;194(1):215–31. doi: 10.1007/s12010-021-03791-7 34988844 PMC8731131

[57] Liu Y, Huang Y, Fan C, Chi Z, Bai M, Sun L, et al. Ursolic Acid Targets Glucosyltransferase and Inhibits Its Activity to Prevent Streptococcus mutans Biofilm Formation. Front Microbiol. 2021;12:743305. doi: 10.3389/fmicb.2021.743305 34646258 PMC8503646

[58] Claesson R, Johansson A, Belibasakis GN. Age-Related Subgingival Colonization of Aggregatibacter actinomycetemcomitans, Porphyromonas gingivalis and Parvimonas micra-A Pragmatic Microbiological Retrospective Report. Microorganisms. 2023;11(6):1434. doi: 10.3390/microorganisms11061434 37374936 PMC10301537

[59] Mallikarjun S, Rao A, Rajesh G, Shenoy R, Pai M. Antimicrobial efficacy of Tulsi leaf (Ocimum sanctum) extract on periodontal pathogens: An in vitro study. J Indian Soc Periodontol. 2016;20(2):145–50. doi: 10.4103/0972-124X.175177 27143825 PMC4847459

[60] Okamura H, Hirota K, Yoshida K, Weng Y, He Y, Shiotsu N, et al. Outer membrane vesicles of Porphyromonas gingivalis: Novel communication tool and strategy. Jpn Dent Sci Rev. 2021;57:138–46. doi: 10.1016/j.jdsr.2021.07.003 34484474 PMC8399048

[61] Sameera U, Paul P, Shivaprasad B, Geetha K. Antimicrobial property of lemongrass oil against Aggregatibacter actinomycetemcomitans. Int J Dent Sci Innov Res. 2019;2:274–84.

[62] Paul P, Sameera U, Geetha K, Bilichodmath S. Antimicrobial property of lemongrass oil against Porphyromonas gingivalis. Can J Dent. 2019;3(1):1–12.

[63] Yoshimasu Y, Ikeda T, Sakai N, Yagi A, Hirayama S, Morinaga Y, et al. Rapid Bactericidal Action of Propolis against Porphyromonas gingivalis. J Dent Res. 2018;97(8):928–36. doi: 10.1177/0022034518758034 29494308

[64] Gow NAR, Hube B. Importance of the Candida albicans cell wall during commensalism and infection. Curr Opin Microbiol. 2012;15(4):406–12. doi: 10.1016/j.mib.2012.04.005 22609181

[65] Gupta A, Jeyakumar E, Lawrence R. Journey of Limonene as an Antimicrobial Agent. J Pure Appl Microbiol. 2021;15(3):1094–110. doi: 10.22207/jpam.15.3.01

[66] Thakre A, Zore G, Kodgire S, Kazi R, Mulange S, Patil R, et al. Limonene inhibits Candida albicans growth by inducing apoptosis. Med Mycol. 2018;56(5):565–78. doi: 10.1093/mmy/myx074 29420815

