# Supplementary material for: Chemical characterization and antimicrobial activities of Citrus aurantifolia peel oils and Ocimum sanctum ethanolic extract
Source: PLoS One. 2025 Sep 5;20(9):e0331710. doi: 10.1371/journal.pone.0331710 (PMC12412941; doi:10.1371/journal.pone.0331710)
Supplement: S1 Table — (DOCX) [file pone.0331710.s001.docx]

**S1 Table** Log₁₀ reduction in time-kill assay of the tested natural agent

| **Oral pathogens** | **Sample Tested** | **Concentration** | **Log_10_ CFU/mL** | | | | | | | | | | | | |
| --- | --- | --- | --- | --- | --- | --- | --- | --- | --- | --- | --- | --- | --- | --- | --- |
|  |  |  | **0 hr** | **2 hr** | **4 hr** | **6 hr** | **24 hr** | **48 hr** | **72 hr** | **∆2hr** | **∆4hr** | **∆6hr** | **∆24r** | **∆48hr** | **∆72hr** |
| ***L. acidophilus*** | Control |  | 5.00 | 5.19 | 5.29 | 5.67 | 7.00 | ND | ND | 0.19 | 0.29 | 0.67 | 2.00 | ND | ND |
|  | Lime TH | 1MIC | 5.00 | 4.47 | 4.72 | 4.60 | 4.46 | ND | ND | -0.53 | -0.28 | -0.40 | -0.54 | ND | ND |
|  |  | MBC | 5.00 | 0.00 | 0.00 | 0.00 | 0.00 | ND | ND | **-5.00** | **-5.00** | **-5.00** | **-5.00** | ND | ND |
|  | Lime SF | 1MIC | 5.00 | 4.34 | 4.47 | 4.53 | 4.08 | ND | ND | -0.66 | -0.53 | -0.47 | -0.92 | ND | ND |
|  |  | 2MIC | 5.00 | 3.90 | 4.51 | 4.48 | 3.87 | ND | ND | -1.10 | -0.49 | -0.52 | -1.13 | ND | ND |
|  |  | MBC | 5.00 | 3.40 | 4.10 | 4.37 | 0.00 | ND | ND | -1.60 | -0.90 | -0.63 | **-5.00** | ND | ND |
|  | OSE | 1MIC | 5.00 | 4.72 | 4.86 | 4.81 | 4.41 | ND | ND | -0.28 | -0.14 | -0.19 | -0.59 | ND | ND |
|  |  | 2MIC | 5.00 | 4.69 | 4.71 | 4.62 | 3.20 | ND | ND | -0.31 | -0.29 | -0.38 | -1.80 | ND | ND |
|  | 0.12% CHX |  | 5.00 | 0.00 | 0.00 | 0.00 | 0.00 | ND | ND | **-5.00** | **-5.00** | **-5.00** | **-5.00** | ND | ND |
| ***S. mutans*** | Control |  | 4.80 | 4.90 | 5.00 | 5.20 | 7.00 | ND | ND | 0.10 | 0.20 | 0.40 | 2.20 | ND | ND |
|  | Lime TH | 1MIC | 4.80 | 4.49 | 4.50 | 4.37 | 4.51 | ND | ND | -0.31 | -0.30 | -0.43 | -0.29 | ND | ND |
|  |  | 2MIC | 4.80 | 4.34 | 4.26 | 4.16 | 3.78 | ND | ND | -0.46 | -0.54 | -0.64 | -1.02 | ND | ND |
|  |  | 3MIC | 4.80 | 4.15 | 4.03 | 3.80 | 3.00 | ND | ND | -0.65 | -0.77 | -1.00 | -1.80 | ND | ND |
|  |  | MBC | 4.80 | 3.78 | 3.60 | 0.00 | 0.00 | ND | ND | -1.02 | -1.20 | **-4.80** | **-4.80** | ND | ND |
|  | Lime SF | 1MIC | 4.80 | 4.55 | 4.66 | 4.47 | 5.00 | ND | ND | -0.25 | -0.14 | -0.33 | 0.20 | ND | ND |
|  |  | 2MIC | 4.80 | 4.26 | 4.34 | 4.31 | 4.15 | ND | ND | -0.54 | -0.46 | -0.49 | -0.65 | ND | ND |
|  |  | 3MIC | 4.80 | 3.92 | 3.92 | 3.94 | 3.20 | ND | ND | -0.88 | -0.88 | -0.86 | -1.60 | ND | ND |
|  |  | MBC | 4.80 | 3.53 | 3.74 | 3.88 | 0.00 | ND | ND | -1.27 | -1.06 | -0.92 | **-4.80** | ND | ND |
|  | OSE | 1MIC | 4.80 | 4.71 | 4.65 | 4.48 | 4.36 | ND | ND | -0.09 | -0.15 | -0.32 | -0.44 | ND | ND |
|  |  | 2MIC | 4.80 | 4.65 | 4.42 | 4.46 | 4.33 | ND | ND | -0.15 | -0.38 | -0.34 | -0.47 | ND | ND |
|  |  | 3MIC | 4.80 | 4.67 | 4.38 | 4.35 | 4.02 | ND | ND | -0.13 | -0.42 | -0.45 | -0.78 | ND | ND |
|  |  | MBC | 4.80 | 0.00 | 0.00 | 0.00 | 0.00 | ND | ND | **-4.80** | **-4.80** | **-4.80** | **-4.80** | ND | ND |
|  | 0.12% CHX |  | 4.80 | 0.00 | 0.00 | 0.00 | 0.00 | ND | ND | **-4.80** | **-4.80** | **-4.80** | **-4.80** | ND | ND |
| ***P. gingivalis*** | Control |  | 5.50 | 5.60 | 5.60 | 5.50 | 5.50 | 6.00 | 7.00 | 0.10 | 0.10 | 0.00 | 0.00 | 0.50 | 1.50 |
|  | Lime TH | 1MIC | 5.50 | 5.00 | 5.00 | 5.00 | 5.00 | 5.00 | 5.00 | -0.50 | -0.50 | -0.50 | -0.50 | -0.50 | -0.50 |
|  |  | 2MIC | 5.50 | 5.00 | 5.00 | 5.00 | 4.90 | 5.00 | 5.00 | -0.50 | -0.50 | -0.50 | -0.60 | -0.50 | -0.50 |
|  |  | MBC | 5.50 | 5.00 | 5.00 | 4.59 | 0.00 | 0.00 | 0.00 | -0.50 | -0.50 | -0.91 | **-5.50** | **-5.50** | **-5.50** |
|  | Lime SF | 1MIC | 5.50 | 5.00 | 5.00 | 5.00 | 5.00 | 5.00 | 5.00 | -0.50 | -0.50 | -0.50 | -0.50 | -0.50 | -0.50 |
|  |  | 2MIC | 5.50 | 5.00 | 5.00 | 5.00 | 4.80 | 4.70 | 5.00 | -0.50 | -0.50 | -0.50 | -0.70 | -0.80 | -0.50 |
|  |  | MBC | 5.50 | 4.60 | 4.60 | 4.70 | 3.70 | 0.00 | 0.00 | -0.90 | -0.90 | -0.80 | -1.80 | **-5.50** | **-5.50** |
|  | OSE | 1MIC | 5.50 | 5.00 | 5.00 | 4.90 | 4.94 | 5.00 | 5.00 | -0.50 | -0.50 | -0.60 | -0.56 | -0.50 | -0.50 |
|  |  | 2MIC | 5.50 | 5.00 | 5.00 | 5.00 | 4.73 | 5.00 | 3.10 | -0.50 | -0.50 | -0.50 | -0.77 | -0.50 | -2.40 |
|  |  | MBC | 5.50 | 5.00 | 5.00 | 5.00 | 4.45 | 4.63 | 0.00 | -0.50 | -0.50 | -0.50 | -1.05 | -0.87 | **-5.50** |
|  | 0.12% CHX |  | 5.50 | 0.00 | 0.00 | 0.00 | 0.00 | 0.00 | 0.00 | **-5.50** | **-5.50** | **-5.50** | **-5.50** | **-5.50** | **-5.50** |
| ***A. actinomycetemcomitans*** | Control |  | 5.00 | 5.26 | 5.12 | 5.48 | 5.88 | ND | ND | 0.26 | 0.12 | 0.48 | 0.88 | ND | ND |
|  | Lime TH | 1MIC | 5.00 | 4.67 | 4.99 | 5.14 | 4.41 | ND | ND | -0.33 | -0.01 | 0.14 | -0.59 | ND | ND |
|  |  | 2MIC | 5.00 | 4.01 | 4.09 | 4.07 | 3.22 | ND | ND | -0.99 | -0.91 | -0.93 | -1.78 | ND | ND |
|  |  | MBC | 5.00 | 0.00 | 0.00 | 0.00 | 0.00 | ND | ND | **-5.00** | **-5.00** | **-5.00** | **-5.00** | ND | ND |
|  | Lime SF | 1MIC | 5.00 | 4.89 | 5.00 | 4.90 | 4.53 | ND | ND | -0.11 | 0.00 | -0.10 | -0.47 | ND | ND |
|  |  | 2MIC | 5.00 | 4.87 | 4.85 | 4.84 | 3.49 | ND | ND | -0.13 | -0.15 | -0.16 | -1.51 | ND | ND |
|  |  | MBC | 5.00 | 3.77 | 4.17 | 3.69 | 0.00 | ND | ND | -1.23 | -0.83 | -1.31 | **-5.00** | ND | ND |
|  | OSE | 1MIC | 5.00 | 5.00 | 5.00 | 5.00 | 3.52 | ND | ND | 0.00 | 0.00 | 0.00 | -1.48 | ND | ND |
|  |  | 2MIC | 5.00 | 5.00 | 4.85 | 4.76 | 3.26 | ND | ND | 0.00 | -0.15 | -0.24 | -1.74 | ND | ND |
|  |  | MBC | 5.00 | 5.00 | 4.50 | 4.10 | 0.00 | ND | ND | 0.00 | -0.50 | -0.90 | **-5.00** | ND | ND |
|  | 0.12% CHX |  | 5.00 | 0.00 | 0.00 | 0.00 | 0.00 | ND | ND | **-5.00** | **-5.00** | **-5.00** | **-5.00** | ND | ND |
| ***C. albicans*** | Control |  | 4.97 | 5.20 | 5.46 | 6.18 | 7.00 | ND | ND | 0.23 | 0.49 | 1.21 | 2.03 | ND | ND |
|  | Lime TH | 1MIC | 4.97 | 4.53 | 3.56 | 3.48 | 4.89 | ND | ND | -0.44 | -1.41 | -1.49 | -0.08 | ND | ND |
|  |  | MBC | 4.97 | 0.00 | 0.00 | 0.00 | 0.00 | ND | ND | **-4.97** | **-4.97** | **-4.97** | **-4.97** | ND | ND |
|  | Lime SF | 1MIC | 4.97 | 5.00 | 5.00 | 5.00 | 4.46 | ND | ND | 0.03 | 0.03 | 0.03 | -0.51 | ND | ND |
|  |  | 2MIC | 4.97 | 3.36 | 3.10 | 0.00 | 3.55 | ND | ND | -1.61 | -1.87 | **-4.97** | -1.42 | ND | ND |
|  |  | MBC | 4.97 | 0.00 | 0.00 | 0.00 | 0.00 | ND | ND | **-4.97** | **-4.97** | **-4.97** | **-4.97** | ND | ND |
|  | 0.12% CHX |  | 4.97 | 0.00 | 0.00 | 0.00 | 0.00 | ND | ND | **-4.97** | **-4.97** | **-4.97** | **-4.97** | ND | ND |

**Notes:** ≥ 3 Log_10_ reduction = Bactericidal effect, < 3 Log_10_ reduction = Bacteriostatic effect

Control: 2% DMSO, 2% Tween in growth medium with oral pathogens

ND: Not determined
